# Clinical predictors of abnormal brain computed tomography findings in mild traumatic brain injury: A cross-sectional study

**DOI:** 10.1097/MD.0000000000034167

**Published:** 2023-06-30

**Authors:** Mehdi Shafie, Mehdi Mahmoodkhani, Iman Salehi, Amin Dehghan

**Affiliations:** a Department of Neurosurgery, Alzahra Hospital, School of Medicine, Isfahan University of Medical Sciences, Isfahan, Iran; b Student Research Committee, Isfahan University of Medical Sciences, Isfahan, Iran.

**Keywords:** computed tomography, mild traumatic brain injury, predictive factor

## Abstract

Mild traumatic brain injury (mTBI) is a health challenge world widely. Local evidence is essential to establish decision-making algorithms. According to the lack of sufficient evidence, the present study aimed to investigate the epidemiology of mTBI and predictive factors of abnormal brain computed tomography (CT) scans. The present analytical cross-sectional study was conducted between March 2021 to September 2022 on patients with the diagnosis of mTBI. Subjects were individuals who were diagnosed with mTBI in 2 Level I trauma centers located in Isfahan province, which serves as the referral center for the entire population of the province. Demographic and clinical data were recorded during a face-to-face interview. The brain CT scans were interpreted by an experienced radiologist. Data were analyzed using IBM SPSS Statistics for Mac, Version 24.0. 498 patients were enrolled in the study, consisting of 393 (78.9%) men and 65 (13.1%) children younger than 10 years old. 100 (20%) of them had abnormal CT scan findings. The mean age of participants was 33.39 ± 19.69, which was significantly higher in patients who had abnormal CT scans (*P* value = .002). Despite the most common mechanism in both groups being motor accidents, the rate of motor accidents was higher in patients with abnormal findings of CT scan (*P* value = .048). Multiple logistic regression revealed that post-traumatic vomiting (PTV) (odd ratios [OR]: 3.736), post-traumatic amnesia (PTA) (OR:3.613), raccoon eyes (OR:47.878), and Glasgow coma scale (GCS) of 15 (OR:0.11) are predictive factors for abnormal findings. The present study suggested the presence of PTV, PTA, raccoon eyes and GCS of 13 or 14 as predictive factors for abnormal findings in mTBI populations.

## 1. Introduction

Traumatic brain injury (TBI) is a worldwide health challenge, resulting in 69 million emergency admissions annually. The overall incidence of TBI was 1299 cases per 100,000 people in North America and 1012 cases per 100,000 people in Europe.^[1]^ However, in developing countries, TBI-related morbidity and mortality are higher.^[2]^

Based on the Glasgow coma scale (GCS) TBI is subdivided into 3 groups: mild (GCS of 13–15), moderate (GCS of 9–12), and severe (GCS below 9).^[3]^ Studies had demonstrated that more severe TBI results in higher rates of morbidity and mortality.^[4–7]^

On the other hand, the most common traumatic central nervous system accident is mild traumatic brain injury (mTBI).^[8]^ Besides, 6% to 21% of patients who suffered from mild TBI (mTBI), have intracranial injuries and 0.4 to 1 percent among them require acute neurosurgery intervention.^[9]^

Brain Trauma Foundation establishes management protocol for mTBI based on the local evidence on the medical center and facilities.^[10,11]^ A patient with mTBI may benefit from the brain computed tomography (CT)^[12]^ scan, though several decision-making algorithms have been produced for performing the CT scan in a patient with TBI, for example, Canadian CT Head Rule^[13]^ and National Institute for Health and Care Excellence.^[11]^ Exposure to X-rays, financial costs, and benefits of the CT scan in the significant number of patients suffering from mild TBI are matters of consideration^[14,15]^; as well as the unavailability of CT scans in remote or deprived areas, especially in developing countries.^[16]^

On the other hand, studies have shown that there is no consensus on predictive factors of traumatic brain lesions and still the CT scan is the gold standard of diagnosis.^[8]^ For example, a study claimed that post-traumatic unconsciousness (PTU) is a strong predictive factor of abnormal brain CT scans^[13]^; while, another study observed no connection between them.^[17]^ Considering that there is no local study on the issue and requires evidence for establishing local guidelines, the present study aimed to investigate the epidemiology of mTBI and predictive factors of the abnormal brain CT scan.

## 2. Methods

### 2.1. Setting

This analytical cross-sectional study was conducted on a total of 498 individuals between March 2021 and September 2022 in Alzahra and Kashani hospitals, 2 Level I trauma centers located in Isfahan province, Iran. Isfahan province has a population of approximately 5.2 million people, and these trauma centers serve as referral centers for the region. The present study was approved by the Research Ethics Committee of Isfahan University of Medical Sciences (IR.MUI.MED.REC.1400.288).

### 2.2. Patients

Subjects were individuals who were diagnosed with mTBI and admitted to the emergency departments of the 2 mentioned hospitals. mTBI was defined as follows.^[18]^

- GCS of 13 to 15 at admission (more than 30 minutes after trauma).
- History of a blow to the head or the presence of a scalp wound, or those with evidence of altered consciousness after a relevant injury.
- No or <30 minutes of loss of consciousness.
- No or <24 hours of post-traumatic amnesia (PTA).

At first, written informed consent was obtained from each participant. Refusing to obtain written informed consent was the exclusion criterion. Also, individuals whose manifestations were attributed to drugs, alcohol, or medications were excluded from the study.

The sample size was calculated to be equal to 500 patients with a refuse rate of 30% based on the Krejcie and Morgan Sample Size Table.^[19]^ To perform probability sampling, 1 day a week was randomly selected each week, and half of the admitted patients on that day were included in the study randomly.

### 2.3. Data collection

Demographic and clinical data were recorded at patients’ hospitalization during a face-to-face interview. Data collectors recorded age, sex, presence of PTA,^[2]^ post-traumatic vomiting (PTV), PTU, post-traumatic seizure, medication-resistant headache, mechanism of trauma, comorbidities and drug history. Data collectors were skillful medical doctors; therefore, examined each patient regarding raccoon eyes, battle signs, rhinorrhea, otorrhea and calculating GCS. Also, the need for emergency neurosurgery intervention was recorded for each patient. The brain CT^[12]^ scans, done for all patients, were reported by an experienced radiologist, who was blinded to clinical presentations and GCS of patients. Brain CT was considered pathological if any of the following conditions were detected: skull base fracture, linear fracture, depressed fracture, subdural hematoma, epidural hematoma, subarachnoid hematoma, contusion, edema, intracranial hemorrhage and intraventricular hemorrhage.

### 2.4. Data management and analysis

Data were analyzed with IBM SPSS Statistics for Mac, Version 24.0. Initially, the normal distribution of continuous data was assessed by the Kolmogorov-Smirnov test. Qualitative and quantitative data are presented as frequency (percentage) and mean ± standard deviation, respectively. Univariate analysis of continuous data was conducted using 2-sample t-tests for normally distributive variables if the homogeneity of variances was confirmed by Leven test. Categorical variables were analyzed using Fisher exact test for variables with at least a cell <5 cases and the Chi-square test for other categorical variables. Statistical significance was defined as *P* < .05. Multiple logistic regression was employed to calculate odd ratios (OR) of potential predictive factors for abnormal CT scans, including as predictors all variables with a *P* value < .25 on univariable test except those variables with a cell containing <5 cases. The Hosmer-Lemeshow chi-square test (*P* value = .784) was used to assess the goodness of fit of the model. Confidence intervals at the 95% level (95% CI) were reported for the ORs.

## 3. Results

During the study, 498 patients participated. Of them, 393 (78.9%) were men. 65 children younger than 10 years old (13.1%) enrolled in the study. Abnormal findings were reported in brain CT scans of 100 participants. Leven test for equality of variances showed the homogeneity of variances in the age variable (*P* value = .988). The mean age of participants was 33.39 ± 19.69, which was significantly higher in patients who had abnormal CT scans (38.83 ± 18.83 vs 32.03 ± 19.68, *P* value = .002). Details are shown in Table 1.

**Table 1:** Clinical characteristics of patients based on CT scan findings and multiple logistic regression of predictive factors for abnormal CT findings.

Variables		Overall (n = 498)	CT findings		*P* value	Odds ratio§ (95% CI)	*P* value
			Abnormal (n = 100)	Normal (n = 398)			
Sex, male, n (%)		393 (78.9%)	89 (89%)	304 (76.4%)	.006*	0.501 (0.212–1.183)	.115
Age, yr, mean±SD		33.39 ± 19.69	38.83 ± 18.83	32.03 ± 19.68	.002†	1.012 (0.995–1.029)	.180
Mechanism, n (%)	Falling	108 (21.7%)	24 (24%)	84 (21.9%)	.002**	-	.239
	Motorcycle accidents	201 (40.3%)	49 (49%)	152 (38.2%)			
	Car accidents	71 (14.3%)	7 (7%)	64 (16.1%)			
	Pedestrian	42 (8.4%)	14 (14%)	28 (7%)			
	Direct trauma	59 (11.8%)	4 (4%)	55 (13.8%)			
	Assault	17 (3.4%)	2 (2%)	15 (3.8%)			
Comorbidities, n (%)	Hypertension	30 (6%)	10 (10%)	20 (5%)	.062*	0.726 (0.175–3.013)	.659
	Ischemic heart disease	11 (2.2%)	3 (3%)	8 (2%)	.468**	-	-
	Diabetes mellitus	19 (3.8%)	3 (3%)	16 (4%)	.777**	-	-
	Cerebrovascular accidents	3 (0.6%)	0	3 (1%)	1.000**	-	-
	Hyperlipidemia	8 (1.6%)	2 (2%)	6 (1.5%)	.664**	-	-
Drug history, n (%)	Anti-coagulants	3 (0.6%)	0	3 (1%)	1.000**	-	-
	Aspirin	23 (4.6%)	12 (12%)	11 (2.8%)	<.001*	3.021 (0.415–22.020)	.275
	Statins	8 (1.6%)	5 (5%)	3 (1%)	.010**	-	-
	Total	43 (8.6%)	29 (29%)	14 (3.5%)	.033*	0.654 (0.146–2.938)	.580
PTV, n (%)		90 (18.1%)	42 (42%)	48 (12%)	<.001*	3.736 (1.873–7.450)	<.001
PTU, n (%)		79 (15.9%)	24 (24%)	55 (13.8%)	.013*	1.131 (0.493–2.594)	.772
PTA, n (%)		79 (15.9%)	38 (38%)	41 (10.3%)	<.001*	3.613 (1.778–7.342)	<.001
PTS, n (%)		0	0	0	-	-	-
Raccoon eye, n (%)		36 (7.2%)	31 (31%)	5 (1.2%)	<.001*	47.878 (15.175–151.059)	<.001
Battle sign, n (%)		0	0	0	-	-	-
Rhinorrhea, n (%)		5 (1%)	5 (5%)	0	<.001**	-	-
Otorrhea, n (%)		0	0	0	-	-	-
Resistant headache, n (%)		52 (10.4%)	14 (14%0	38 (9.5%)	.193*	1.666 (0.707–3.926)	.243
GCS, n (%)	15	469 (94.2%)	72 (72%)	397 (99.7%)	<.001**	0.110 (0.028–0.430)	.002
	14	14 (2.8%)	14 (14%)	0		-	-
	13	15 (3%)	14 (14%)	1 (0.3%)		-	-
Candidate for surgery, n (%)		16 (3.2%)	16 (16%)	0	<.001**	-	-

CT = computed tomography, GCS = Glasgow coma scale, PTA = post-traumatic amnesia, PTS = post-traumatic seizure, PTU = post-traumatic unconsciousness, PTV = post-traumatic vomiting.

* Chi-square test.

** Fisher exact test.

§ Multiple logistic regression included all variables with values in the last 2 columns.

† Independent *t* test.

As Table 1 shown, mechanisms of trauma were significantly different between groups (*P* value = .002). Though the most common mechanism in both groups was motor accidents, the rate of motor accidents was higher in patients with abnormal findings of CT scan (49% vs 38.2%, *P* value = .048). The most common comorbidities were hypertension (6%) and diabetes mellitus (3.8%). There was no statistically significant difference between groups regarding comorbidities. Aspirin was the most used drug in the population and also analysis revealed that aspirin use is much higher in patients with abnormal brain CT scans (12% vs 2.8%, *P* value < .001). Analyses showed that the use of statins and positive drug history, in general, were higher among patients with abnormal findings.

Neither case of battle signs nor otorrhea was found in the population. However, raccoon eyes (31% vs 1.3%) and rhinorrhea (5% vs 0%) were mostly detected in patients with abnormal CT scans (both *P* values < .001). Besides, PTA (38% vs 10.3%, *P* value < .001), PTV (42% vs 12%, *P* value < .001) and PTU (24% vs 13.8%, *P* value = .013) were more common among patients with abnormal CT scans.

**Figure 1.**
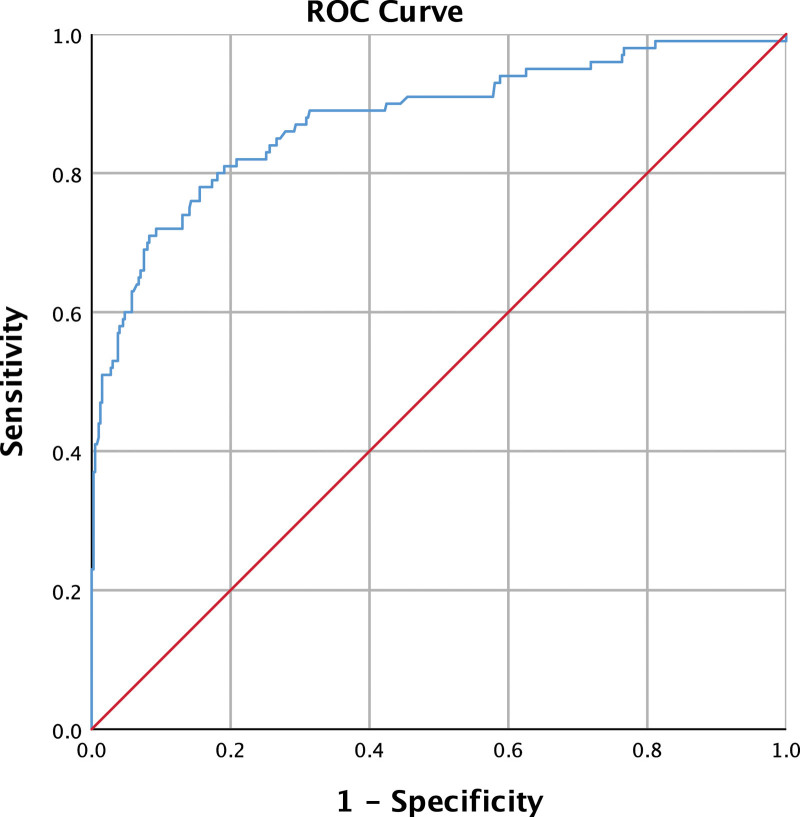
ROC curve analysis of the multiple logistic regression on predictive factors of abnormal brain CT scans findings. The area under ROC curve = 0.877, 95% confidence interval: 0.834 to 0.921. CT = computed tomography, ROC = receiver operating characteristic.

Results from the logistic regression analysis are exhibited in Table 1. Analyses revealed the PTV [OR:3.736], PTA [OR:3.613] and Raccoon eyes [OR:47.878] are predictive factors for abnormal findings in mTBI populations. However, a GCS of 15 [OR:0.110] is revealed to decrease the probability of abnormal findings in brain CT scans. The sensitivity, specificity, positive predictive value (PPV) and negative predictive value of the predictive model are 53%, 96.7%, 80% and 89.12%, respectively. Receiver operating characteristic curve analysis of the multiple logistic regression on predictive factors of abnormal brain CT scan findings is shown in Figure 1.

Eventually, 16 patients with abnormal brain CT scans were candidates for emergency brain surgery (Table 2). The most common diagnoses for emergency surgery were epidural hematoma (10 patients), followed by subdural hematoma (5 patients) and skull base fracture (1 patient).

**Table 2:** Classification of intracranial pathology in patients with abnormal CT findings and candidates for emergency surgery.

	Pathology	Abnormal CT findings (n = 100)
Abnormal findings of Brain CT scans, n (%)	Skull base fracture	32 (32%)
	Linear fracture	27 (27%)
	Depressed fracture	17 (17%)
	Subdural hematoma	16 (16%)
	Epidural hematoma	12 (12%)
	Subarachnoid hematoma	11 (11%)
	Contusion	9 (9%)
	Edema	3 (3%)
	Intracranial hemorrhage	2 (2%)
	Intraventricular hemorrhage	1 (1%)
Causes of emergency brain surgery, n (%)	Epidural Hematoma	10 (10%)
	Subdural hematoma	5 (5%)
	Skull base fracture	1 (1%)

CT = computed tomography.

## 4. Discussion

The present study investigated the relationship between clinical presentations and brain CT scans finding of patients with mTBI. This study reported that distribution of age, sex, mechanisms of trauma, use of statins and aspirin and presence of PTV, PTU, PTA, raccoon eyes and rhinorrhea were significantly different among patients with abnormal findings in brain CT scans compared to them without any abnormality. Also, multiple regression exhibited that the presence of PTV, PTA, raccoon eyes and a GCS of 14 or 13 are predictive factors for abnormal findings in mTBI populations. Although, the analysis showed a GCS of 15 could be a predictive factor for a normal brain CT scan.

Annually, more than 600 per 100,000 people suffer from a mTBI, and the most common mechanisms of trauma are falls or vehicle collisions.^[12,20]^ Also, the risk of mTBI for men is 2-fold for women.^[12]^ Regardless of proven the long-term effects,^[21,22]^ mTBI applies enormous costs and devastating conditions to individuals and the health system. Intracranial pathologies are unusual (6%–21%); however, 0.4% to 1.0% of cases with mTBI require prompt neurosurgical interventions.^[9]^ Besides, the availability of medical facilities and healthcare providers is essential for the proper diagnosis and management of mTBI. Therefore, numerous brain trauma Foundations established evidence-based guidelines to improve the quality of care, facilitate medical practice, especially in remote areas, and cut unnecessary costs.

Up to now, numerous studies investigated clinical factors associated with intracranial pathology and developed models and algorithms for the use of CT scan in patients with mTBI. A study by Turedi et al on 240 patients with mTBI found abnormal brain CT scan findings in 19% of them.^[17]^ They reported that 59% and 86% of patients with GCS of 14 and 13 had abnormal CT scans. Also, they suggested that PTV [OR:4.61, 95%CI:2.20–9.64] was a predictive factor for the abnormal brain CT scan. However, PTA, PTU, headache and multi-trauma were not valuable to predict abnormal brain CT scans. Another study by Nee et al suggested PTV as a strong predictive factor for intracranial pathology.^[23]^ A prospective cohort study on 3121 patients by Stiell et al recommended PTV, GCS below 15, open or depressed skull fracture, signs of base skull fracture, older than 65 years old, PTA and PTU as predictive factors for important brain injury.^[13]^ The present study is in line with Turedi, Nee and Stiella studies regarding suggesting PTV as a predictive factor. This study also is in line with Stiell et al but is in contrast with Turedi et al regarding considering PTA as a predictive factor. Besides, the present study and Stiell study both suggested GCS below 15, PTA, PTU and raccoon eyes as predictive factors. Our analyses did not reveal age as a predictive factor, in contrast to Stiell study.

A study by Pages et al on 500 patients aged 65 and over found that 7.6% of them had traumatic brain lesions.^[24]^ They also proposed male sex, PTU and focal neurological deficit as related factors to the presence of lesions in brain CT scans. A study by Nayebaghayee et al reported that 27% of patients with a GCS of 15 required emergency brain surgery.^[8]^ Also, they found that the most common traumatic brain lesion was epidural hematoma (38.5%), followed by cerebral contusion (29.4%), and pneumocephaly (17.4%). They concluded that GCS was not enough accurate to distinguish traumatic brain lesions and the CT scan still was the gold standard to determine traumatic brain lesions. Literature showed that there is controversy on considering PTU as a predictive factor. It might have risen from that history taking in emergency departments is a complex task and sometimes patients cannot provide accurate history, especially in cases of high-energy trauma in which patients were shocked. Therefore, it seems to be an important question to answer in further studies. Our study also did not support the consideration of the male sex as a predictive factor. Though the present study suggested GCS as a predictive factor, we agree with Nayebaghayee study on the inability to fully rely on GCS, instead of other predictive factors and CT scan findings.

Regarding PTV, Turedi, Nee and Stiell studies and our findings suggest it as a predictive factor. In addition, Nee et al concluded that there was no difference between a single episode and multiple episodes of vomiting. Findings in PTA are still controversial; Stiell study and our findings support PTA as a predictive factor; however, Turedi study opposed it. Relying on GCS alone was shown to be wrong in Nayebaghayee study. Although, a GCS of 15 is suggested as a protective factor for abnormal CT scan findings based on Nayebaghayee study and our results.

## 5. Limitations

We had some limitations. First, we enrolled 100 patients with abnormal CT findings. It seems worthwhile if future studies investigated these issues in larges longitudinal study designs. Despite including 2 main trauma centers of Isfahan Province in this study, some patients might refer to different trauma centers. Thus, it is strongly suggested that further studies conduct based on registry systems, in which none of the traumatic patients is left out of the study.

## 6. Conclusion

The present study suggested the presence of PTV, PTA, raccoon eyes and GCS of 13 or 14 as predictive factors for abnormal findings in mTBI populations.

## Acknowledgments

The team would like to express their gratitude towards whom helped us in data gathering and coordination, including Ms. Sheibani, Dr Niloofar Javadi, Dr Arezoo Khalili and Dr Amirhosein Ghandehari.

## Author contributions

**Conceptualization:** Mehdi Shafie, Mehdi Mahmoodkhani.

**Investigation:** Iman Salehi, Amin Dehghan.

**Methodology:** Mehdi Shafie, Mehdi Mahmoodkhani, Amin Dehghan.

**Project administration:** Amin Dehghan.

**Supervision:** Mehdi Shafie.

**Visualization:** Amin Dehghan.

**Writing – original draft:** Amin Dehghan.

**Writing – review & editing:** Mehdi Shafie, Mehdi Mahmoodkhani, Iman Salehi, Amin Dehghan.
